# Impact of Psychiatry Posting on Attitudes Toward Mental Illness: A Prospective Cohort Study of Medical Students From Three Universities

**DOI:** 10.1177/23821205241275420

**Published:** 2024-08-19

**Authors:** Oluyemi O Akanni, Imesidayo O Eboreime-Oikeh, Oderinde K Oyeyemi, Anthony A Olashore

**Affiliations:** 1 Clinical Services, Federal Neuropsychiatric Hospital, Benin City, Edo State, Nigeria; 2 Department of Medicine, College of Health Sciences, Igbinedion University, Okada, Nigeria; 3 Department of Mental Health, 251350 University of Benin Teaching Hospital, Benin City, Nigeria; 4 Department of Psychiatry, Faculty of Medicine, 54547 University of Botswana, Gaborone, Botswana

**Keywords:** Attitudes, medical students, mental illness, Nigeria

## Abstract

**INTRODUCTION:**

Negative attitudes towards mental illness are known to exist among medical students in Nigeria. However, the effect of undergoing a rotation in psychiatry on their attitudes is uncertain.

**OBJECTIVES:**

It aimed to determine the effect of psychiatry posting during medical training on medical students’ attitudes toward mental illness and examine the association of posting-related and demographic factors with their attitudes.

**METHODS:**

It is a prospective cohort study in which 187 medical students from three different universities were followed up during their psychiatry postings in two different posting sites. Questionnaires containing basic demography, posting-related variables, and the Community Attitude to Mental Illness (CAMI) scale were administered to all available students before and after the rotation in psychiatry. A paired t-test was applied to test the differences in the CAMI scores before and after posting, while ANOVA and hierarchical regression were utilized to determine the association of variables with the CAMI scores.

**RESULTS:**

There were significant differences between the pre-and post-rotation CAMI scores in CAMI's social restrictiveness (SR) and community mental health ideology (CMHI) domains, indicating improved attitudes. Demographic factors had no significant influence on the students’ attitudes, but the length of posting, university of the students, and posting sites had varying impacts.

**CONCLUSION:**

Psychiatry rotation has a positive impact on students’ attitudes toward mental illness in both posting sites, particularly in the domains of SR and CMHI. This underscores the importance of psychiatry training in medical education in changing the attitudes of future healthcare professionals.

## Introduction

Mental illness is a growing public health issue with disturbing rates of stigmatization and discrimination.^1^ Though levels of mental health literacy (MHL) in the general population seem to be increasing, negative attitudes to and stigmatization of the mentally ill have not improved in tandem with MHL.^2,3^ Hence, attitude may not always reflect knowledge. This is further confirmed in cases of healthcare professionals and mental health trainees who have been found to possess negative attitudes towards mental illness and those who suffer from it.^4–7^ The stigmatizing attitudes of medical students and health professionals towards mental illness can hinder the quality of care that individuals living with mental health disorders receive. This could invariably have a negative impact not just on their mental health but also on their overall well-being.

Personal experience, culture, stereotypes, and information influence perceptions and attitudes.^8^ A study among medical students in Nigeria demonstrated significantly improved attitudes toward mental illness after a mental health educational intervention.^7^ Besides mental health education interventions, researchers are increasingly interested in how psychiatry rotation or posting affects medical students’ attitudes toward mental illness, even if the posting doesn't have a specific anti-stigma component.^9^

Numerous studies have been conducted to determine the influence of psychiatry training on medical students’ attitudes toward mental illness, both within Nigeria^7,10–12^ and outside the country.^9,13–19^ The majority of these studies suggest that attitudes toward patients with mental illness improve, and the students become more tolerant post-psychiatry rotation.^9,14–18,20^ Although there is a wealth of available data, there is a need for ongoing research to enhance understanding of how psychiatry posting shapes positive attitudes and eliminates negative attitudes among medical students towards individuals with mental illness.

Low- and middle-income countries (LMICs) continue to bear 70% of the global burden of mental disorders, and research studies indicate that attitudes tend to be more negative and stigmatizing in these countries.^21–23^ This underscores the need for local research in LMICs. In Nigeria, the available data is sadly skewed; the majority of the studies reported in the literature have come from the South-West region of the country.^7,10–12^ Other regions either lack or are deficient in studies on this aspect. One of the few studies conducted in the South-South region among medical students was carried out in a single center.^20^ Using the modified version of the World Psychiatric Association questionnaire developed for a program to reduce stigma and discrimination, the study found a change in stigmatizing and discriminatory attitudes in the students’ attitudes toward mental illness following an 8-week psychiatry rotation. However, the primary focus of the study was on the changes in the understanding of the cause of mental illness rather than attitudinal change.

The present study was therefore conducted to bridge the gap in knowledge in the South-South region of the country. It compared the attitudes of medical students from South-South Nigeria towards mental illness before and after their psychiatry posting to determine the impact of the rotation on their attitudes. It also aimed to determine the factors associated with their attitudes, including posting-related and sociodemographic factors such as age, gender, marital status, and religion. The study was conducted among medical students from three universities undergoing psychiatry postings in two training centers. Though it was hypothesized that exposure to psychiatry would positively alter the students’ attitudes towards mental health disorders, as it would offer them first-hand experience and increased understanding, it remains unclear whether the university of training and posting site of students have a significant impact on their attitude toward mental illness. Moreover, while extant research suggests that female students exhibit a more compassionate attitude towards mental illness than their male counterparts,^5,8,9,24^ other demographic factors that may influence attitude have not been adequately explored or have yielded inconsistent results;^4,9,10,25^ thus, we posited a null hypothesis that these socio-demographic variables are not determinative of attitudes toward mental illness.

## Methods

### Study design

This prospective study, conducted at multiple centers, compared participants’ attitudes before and after completing a psychiatry posting, and its reporting conforms to the Strengthening the Reporting of Observational Studies in Epidemiology (STROBE) statement.^26^

### Study location

The study was conducted at two posting sites involving medical students from three universities in South-South Nigeria. The first site was the University of Benin Teaching Hospital (UBTH), where medical students from the University of Benin (UNIBEN) do all their clinical postings, including psychiatry. UBTH is a tertiary hospital that offers specialist services and a general practice unit that provides primary care services. It also offers excellent opportunities for medical education at both the undergraduate and postgraduate levels in Nigeria. The Mental Health Department at UBTH offers inpatient care with 30 beds for admission and outpatient clinics that operate twice a week. On average, 30 individuals attend each clinic session.

The second study site was the Federal Neuropsychiatric Hospital, Benin City (FNPHB), located in South-South Nigeria. It is a stand-alone specialist service center that provides mental health care services and also runs a general medical practice clinic. The hospital offers postgraduate training for resident doctors and rotations in psychiatry for medical students. This is where students from Igbinedion University Okada (IUO) and Edo State University, Uzairue (ESUU) undertook their rotations in psychiatry. The FNPHB is a 260-bed facility with an occupancy rate of over 90%. It provides specialized inpatient and outpatient care, including emergency psychiatric services. The outpatient clinic operates four times a week, with an average attendance of 140 patients per clinic.

### Study population

This study was conducted among medical students from the three previously mentioned universities. In Nigeria, the undergraduate medical program spans six years. The first three years of study are the preclinical phase, while the last three years are the clinical phase of training, which exposes the students to clinical situations and is largely done in a hospital setting. The medical students were from the sixth year UNIBEN (a federal institution), the fifth year IUO (a privately owned facility), and the sixth year ESUU (a state government-run university). All the participants were in their clinical phase of training with no background training in psychiatry. As part of the medical training, students are required to undertake a posting in psychiatry. The Nigerian University Commission recommends a minimum of 6 weeks of exposure to psychiatry for medical students.

### Posting

The UNIBEN students attended a 7-week psychiatry posting at the Mental Health Department of UBTH from August 8 to September 26, 2022. During their posting, they received didactic lectures, case demonstrations, and clinical seminars. Also, the students interacted with the patients by taking medical histories and conducting examinations before discussing them with the consultants. The posting ended with an assessment that tested their knowledge of psychiatry.

The IUO medical students spent 12 weeks posting between July 13 and October 7, 2022, at the FNPHB. The posting consisted of an initial two weeks of didactic lectures covering the curriculum, followed by nine weeks of clinical rotation within the six subspecialty units in the hospital. During the clinical posting, the students participated in consultants’ clinics and ward rounds and observed electroconvulsive therapy and psychotherapy sessions. They also received bedside teachings and tutorials. Additionally, they are obligated to complete a minimum number of clerkships, examinations, and presentations of cases in various settings, including the emergency unit, the outpatient clinic, and the ward round. The last week of their rotation was utilized for their end-of-posting examination.

The ESUU students had a 7-week rotation at the FNPHB from August 30 to October 21, 2022. Their exposure was similar to that of IUO students, except that they had lectures run concurrently with their clinical posting. The lectures took place in the first 2 hours of resumption of activity for the day, while clinical posting took place afterward. The last week of the posting was also reserved for the end-of-posting examination.

### Sampling and selection process

All the medical students from the three universities freely and voluntarily gave consent and were selected for the study. It was a survey involving all the medical students on mental health posting from the three universities. The total number of students was 187, comprising 82 UNIBEN, 64 IUO, and 41 ESUU students.

### Selection criteria

The inclusion criteria are medical students who were on mental health posting and were willing to participate in the study. Those who failed to provide consent were excluded.

### Instruments

The following were used for the study.

**The Sociodemographic and Posting-Related Data Collection Sheets** were specifically designed to inquire about the students’ sociodemographic characteristics, including gender, age, marital status, and religion. They also contained posting-related factors such as the posting site, the university of training, the posting duration, and the medical students’ levels.

**Community Attitudes Towards Mental Illness Questionnaire:** This was used to obtain the students’ negative and positive attitudes toward mental illness and mentally ill persons. It is a 40-item self-report scale that is widely used and has been proven reliable and valid.^27^

The scale has four dimensions: authoritarianism, which corresponds to a view of the mentally ill as someone inferior who needs coercive handling; benevolence, which refers to a paternalistic view of the mentally ill; social restrictiveness (SR), which concerns the belief that the mentally ill are a threat to society and should be avoided; and community mental health ideology (CMHI), which indicates the rejection of mental health services and mentally ill persons in the community. Each sub-scale has ten items, and the subscales are all rated according to a 5-point Likert scale ranging from strongly agree to strongly disagree. The minimum and maximum scores for each CAMI subscale were 5 and 50, respectively. Higher scores on the CAMI subscales indicate more negative attitudes. The alpha coefficients obtained in the study were 0.46, 0.70, 0.70, 0.76, and 0.83 for the authoritarianism, benevolence, social restrictiveness, community mental health ideology subscales, and the total scale, respectively. This report is similar to the conclusion drawn in a systematic review of studies taken over 40 years; the construct validity and reliabilities of the CAMI scale and subscales are acceptable, with the authoritarian subscale being the weakest.^27^

### Ethical consideration

The study's application for approval was obtained from the Ethics and Research Committee of the University of Benin Teaching Hospital, Benin City (Ref no: ADM/E 22/A/VOL. VII/14830112959). Written informed consent was also obtained from every respondent who agreed to participate in the study.

### Procedure

The questionnaire was presented to the students at the resumption and the end of their psychiatry posting. It took 10 min or less for a respondent to fill out the questionnaire**.** They were verbally informed of the content and nature of the study and were assured that participation was voluntary and non-participation would not attract any consequences. Additionally, anonymity was observed when completing the questionnaires. Data were collected by two investigators who work at the sites. All the pre-posting questionnaires were obtained within 72 hours of the commencement of posting. Most of the collection was done on the first day when the students had their orientation of the posting; however, the collection was extended beyond the first day to ensure a census sampling. The post-rotation questionnaire was administered at the venue of the end of the posting assessment, where all the students were present.

### Data analysis

The data collected was analyzed using the Statistical Package for Social Sciences (SPSS) version 26 (SPSS for Windows 26, SPSS Inc., Chicago, IL, USA). Descriptive statistics such as frequencies, means, and standard deviations were used. A normality test of the attitudes score was done, and it showed a normal distribution. A chi-square was used to determine the differences in the categorical variables of the gender, religious affiliation, and marital status of the students, while analysis of variance (ANOVA) was utilized to determine the differences in their mean ages. A paired t-test was used to analyze the difference between the CAMI scores before and after posting. ANOVA was again applied to determine the differential impact of the three universities on the two CAMI subscales [social restrictiveness (SR) and community mental health ideology (CMHI), which showed significant change following the psychiatry rotation.

Furthermore, a 2-step hierarchical multiple regression was carried out to determine the independent effects of the university on SR and CMHI. Age, gender, and religion were first entered into the model to control their effect on SR and CMHI; thereafter, the university of training was entered in Step 2. All the analysis was set at *P* < .05 for the significance level.

## Results

Table 1 shows there were more males (53%) than females (47%) overall, though the females outnumbered the males in IUO and ESUU. All the students’ mean age was 24.18 (SD 3.49) years. Students from UNIBEN had the highest mean age [25.73 (3.02) years]. The majority of the participants were single (90.3%) and Christians (86.5%). There were statistically significant differences in the students’ sociodemographic characteristics across the universities, except for marital status.

**Table 1.** Socio-demographic characteristics of students from the 3 universities.

POSTING SITE	FNPHB		UBTH		
UNIVERSITY VARIABLES	IUO	ESUU	UNIBEN	TOTAL	SIGNIFICANT TEST
Gender*					X^2 ^= 20.7
Male	28 (44.4)	12 (30.0)	58 (70.7)	98 (53.0)	*P* < .001
Female	35 (55.6)	28 (70.0)	24 (29.3)	87 (47.0)	
**Age**					F = 18.4
Mean (SD)	22.57 (3.32)	23.46 (3.34)	25.73 (3.02)	24.18 (3.49)	*P* < .001
Marital status*					X^2 ^= 1.46
Single	60 (93.8)	36 (90.0)	72 (87.8)	168 (90.3)	*P* = .48
Others^	4 (6.3)	4 (10.0)	10 (12.2)	18 (9.7)	
Religion*					X^2 ^= 10.5
Christianity	55 (85.9)	29 (72.5)	76 (93.8)	160 (86.5)	*P* = .01
Others^#^	9 (14.1)	11 (27.5)	5 (6.2)	25 (13.5)	

Abbreviations: FNPHB, Federal Neuropsychiatric Hospital, Benin; UNIBEN, University of Benin; UBTH, University of Benin Teaching Hospital; IUO, Igbinedion University Okada; ESUU, Edo State University, Uzairue.

*Missing value. ^Married, divorced, separated. # Traditional and Islam.

Table 2 shows that scores in the CAMI scale and subscales were lower after the psychiatry posting, except for the benevolence subscale, where scores were higher after the posting than before. However, the change in CAMI subscale scores from before posting to after posting was only significant for SR and CMHI.

**Table 2.** Comparison of CAMI scores before and after posting.

CAMI	BEFORE POSTING		AFTER POSTING		MEAN DIFFERENCE	95%		SIGNIFICANT TEST		
	MEAN	SD	MEAN	SD		LOWER	UPPER	*t*	df	*P*
**Authoritarian**	27.2	4.88	26.3	4.96	0.89	−0.08	1.87	1.82	180	.071
**Benevolence**	21.1	4.94	21.6	6.49	−0.55	−1.75	0.65	−0.09	177	.365
**SR**	24.9	5.28	23.5	6.11	1.46	0.33	2.60	2.54	182	.012
**CMHI**	26.4	5.48	25.2	6.60	1.20	0.06	2.34	2.07	183	.040
**Total**	100.1	15.1	96.9	18.8	3.16	−0.45	6.77	1.73	164	.085

Abbreviations: CAMI, community attitudes towards mental illness; SR, social restrictiveness; CMHI, community mental health ideology.

A 1-way between-groups analysis of variance was conducted to explore the relationship between the university of training and the post-rotation scores of SR and CMHI. There was a statistically significant difference in the SR [F (2, 182) = (9.73), *P* < .001] and CMHI [F (2, 181) = 9.61, *P* < .001] scores for the three universities. The effect size, calculated using eta squared, was moderately large (0.10) for both SR and CMHI; this value falls within the range of moderate (0.06) and large (0.14) effect sizes proposed by Cohen.^28^ Post-hoc comparisons using the least significance difference (LSD) test indicated that the mean score for UNIBEN (which was the highest) was significantly different from the mean scores for both IUO (which was the least) and ESUU, while there was no significant difference between the IUO and ESUU for both SR and CMHI scores (Table 3).

**Table 3.** One-way ANOVA showing the effect size with LSD post-hoc Tests.

CAMI SUBSCALE		SS	F	*P*	UNIVERSITY COMPARISON	MEAN DIFFERENCE	STD ERROR	*P*
**CMHI**	Btw grp	765.37	9.61	<.001	IUO - UNIBEN	−4.647	1.065	<.001
	Within grp	7208.98			ESUU - UNIBEN	−2.479	1.210	.042
	Total	7974.34			IUO - ESUU	−2.168	1.270	.090
**SR**	Btw grp	662.34	9.73	<.001	IUO - UNIBEN	−4.241	0.978	.000
	Within grp	6195.69			ESUU - UNIBEN	−2.733	1.121	.016
	Total	6858.02			IUO - ESUU	−1.508	1.167	.198

Abbreviations: SS, sum of squares; SR, social restrictiveness; CMHI, community mental health ideology; CAMI, community attitudes towards mental illness.

Following the outcome of this post-rotation analysis, we conducted another post hoc ANOVA analysis on the differences in the SR and CMHI scores pre-rotation to determine whether differences noted across the universities existed before posting. There was no significant difference in the pre-rotation CAMI scores of students from the three universities (the result is not tabulated).

In Table 4, hierarchical multiple regression was employed to determine the independent impact of the student's university on their post-rotation SR and CMHI levels while controlling for the influence of age, gender, and religion. In Model 1, age, gender, and religion were considered, but they were found to be insignificant in explaining the variance in the CMHI and SR domains, accounting for only 1.3% and 2.5% of the variance, respectively. After the entry of the training center in Model 2, the total variance explained by the model increased modestly to 9.3% and 7.4% in the CMHI and SR domains, respectively. The contributions of CMHI and SR to the variance in attitude after controlling for age, gender, and religion were statistically significant (*P* < .001).

**Table 4.** Hierarchical regression analysis for age, gender, religion, and university (training center) predicting social restrictiveness and community mental health ideology.

CAMI SUBSCALE		MODEL 1			MODEL 2		
	PREDICTORS	β	*P*-VALUE		β	*P*-VALUE	
**CMHI**	Age	0.016	.843	R^2^ Δ = 0.013	−0.104	.202	R^2^ Δ = 0.093
	Gender	−0.075	.347	F Δ = 0.754	−0.031	.686	F Δ = 18.381
	Religion	−0.081	.284	*P* = .522	−0.045	.533	*P* < .001
	Center				0.339	<.001	
**SR**	Age	−0.087	.272	R^2^ Δ = 0.025	−0.019	.811	R^2^ Δ = 0.074
	Gender	−0.067	.399	F Δ = 1.552	−0.028	.720	F Δ = 14.553
	Religion	−0.097	.193	*P* = .203	−0.066	.365	*P* < .001
	Center				0.302	<.001	

Abbreviations: R^2^ Δ, R squared change; F Δ, F change; β, standardized regression coefficient; SR, social restrictiveness; CMHI, community mental health ideology.

## Discussion

The present study set out to determine the impact of psychiatry posting during medical training on the attitudes of medical students toward mental illness. Additionally, it intended to investigate the association between students’ demographic and posting-related factors and their attitudes toward mental illness. Negative attitudes towards mental illness are widespread in society, leading to stigmatization and discrimination against individuals living with mental illness. Despite their education and training, this is particularly true even among healthcare professionals and trainees. Unfortunately, mental health literacy has not been consistently shown to improve attitudes towards mental illness.^3,8,29^

The present study found evidence of a positive impact of some weeks of psychiatry posting on the attitudes toward mental illness of the medical students in all three universities. This finding is consistent with prior research conducted in Southwest Nigeria,^9,11,12^ which also reported a favorable change in trainees’ attitudes toward mental illness following their exposure to related training programs. While the current study did not evaluate the long-term sustainability of the positive attitudinal shift, previous research has demonstrated that the positive change in attitude can be long-lasting.^30,31^ This finding suggests that psychiatry posting can serve as an effective method of fostering positive attitudes among medical students toward mental illness even when the training does not include a focused anti-stigma input.

Worthy of note is the change in attitude to mental illness and the mentally ill found in this study was only significant for two of the four domains of the Community Attitudes to Mental Illness (CAMI) instrument. The two domains where positive attitudinal change was significant after the psychiatry posting were Social Restrictiveness (SR) and Community Mental Health Ideology (CMHI). Furthermore, the change in attitude was modest, even after controlling for age, gender, and religion. These findings could imply that the medical students’ perception of the threat posed by the mentally ill to society was significantly less after their psychiatry training compared to their previously held beliefs. Similarly, the outcome of this study suggested that students were significantly more likely to embrace or support mental health services within the community after their exposure to psychiatry than before the training. Despite some improvements in the Authoritarianism domain, the Benevolence domain has decreased. Unfortunately, the changes in attitude of the Benevolence and Authoritarianism domains were not remarkable. Nonetheless, negative attitudes across all four domains of CAMI must improve because each domain contributes to societal perceptions and behaviors, including stigmatization and discrimination.^32^

The study found that before undergoing psychiatry training, students’ attitudes toward mental illness did not vary significantly across the universities. However, following the psychiatry posting, a discrepancy emerged in the attitudes of the students from different universities, with students from one university exhibiting a more significant change in attitude than those from other universities. The reason for the difference between the universities is unclear, but it is worth noting that the students from IUO who had the longest posting duration exhibited the greatest attitudinal change in all the CAMI domains. This may imply that a more extended training period may be a key factor in promoting greater attitudinal change.

Besides posting duration, other factors must have contributed to the attitudinal differences, as significant variations existed between UNIBEN and ESUU, two universities with identical posting lengths. Upon careful review, it was observed that the degree of positive attitudinal change was significantly higher among students from IUO and ESUU, who had their psychiatry training at FNPHB, compared to those from UNIBEN who did their posting in UBTH. This suggests that differences in institutional environment, clinical exposure, psychiatric instructional capacity, and focus during training may have played a role. For example, the clinical exposure at the FNPHB provided the medical students with greater opportunities for patient interaction due to the institution's high patient volume compared to UBTH. A systematic review has shown that contact of medical students with mentally ill patients leads to attitudinal change.^33^

The present study did not find significant predictive effects of any of the socio-demographics on the SR or CMHI attitudes during regression. Nonetheless, some other studies found evidence that indicated the female gender affects attitude toward mental illness more positively than the male.^4,9,24,25^ The effect of gender may be expected, considering that females tend to be more empathetic, caring, and tolerant. On the other hand, the research finding of the impact of age on the attitude of medical students is variable; while a study found younger age to be associated with better attitudes,^10^ another study did not report age to have any impact.^9^ The lack of a relationship between socio-demographics and attitude in this study implies that age, gender, or religion may not be a focus for intervention in attitudinal change programs among the students.

The findings of this study must be considered within the context of several limitations. Firstly, the use of the CAMI scale as a self-report instrument to assess attitudes introduces the possibility of social desirability bias. The bias may have influenced students’ responses, potentially skewing them to be viewed favorably by their lecturers, who were also the investigators. Secondly, despite being a prospective study, the relatively short period between pre- and post-posting questionnaire applications may have favored positivity due to the recollection of recently taught material. Consequently, the enduring effect of knowledge and training may not have been fully captured. Additionally, the use of samples consisting of students from three universities in the same state within the South-South region of Nigeria may limit the generalizability of the study's outcomes to other contexts. However, the inclusion of students from Private, State, and Federal universities ensured diversity within the sample population. 

Despite these limitations, the study possessed notable strengths. The relatively large sample size enabled reasonable inferences to be drawn from the data. Also, the multi-center design facilitated the analysis of institutional differences, allowing for a richer understanding of how diverse settings impact shifts in attitude among medical students. Furthermore, the CAMI instrument utilized in the study has been shown to have internal consistency, validity, and temporal stability.^34^ These strengths enhance the credibility and reliability of this study's findings.

## Conclusion

In conclusion, this study found a significant improvement in medical students’ attitudes toward mental illness following their psychiatry posting. This positive change was consistent with previous research conducted in various countries, supporting the effectiveness of psychiatric training in improving attitudes towards mental illness. The attitudinal change was not affected by sociodemographic factors such as age and gender.

However, there were differences in the magnitude of attitude change among students from different universities and posting centers, suggesting potential variations in educational approaches or institutional environments. Although the positive change in attitude was particularly significant in domains related to reducing social restrictiveness and promoting community mental health ideology, there remains room for improvement in other domains, such as authoritarianism and benevolence. Addressing these domains is crucial for combating the stigma and discrimination associated with mental illness. Moreover, we advocate a training model for medical students that prioritizes maximal patient contact and interaction. This approach has proven to be an effective tool in promoting empathy and breaking down stigmatizing attitudes. Further, we recommend that medical training programs ensure sufficient psychiatry posting duration to allow the medical students to cultivate and maintain positive attitudes because the length of psychiatry posting influences the extent of attitudinal change. The development of a positive and tolerant attitude among medical students is essential for providing high-quality mental health care.

Overall, this research underscores the importance of integrating psychiatry training into medical education to promote positive attitudes toward mental illness among future healthcare professionals. Continued efforts are needed to enhance mental health literacy and to specifically adopt anti-stigma reduction strategies in mental health training programs to ultimately improve the quality of care for individuals living with mental illness. A large, multi-center prospective cohort study with a longer follow-up is recommended to resolve any inconsistencies in the enduring impact and relevance of mental health intervention on the effectiveness of care, elimination of stigmatization, and overall well-being of the mentally ill in society.
